# The association of socioeconomic status and response to pediatric health behavior and lifestyle obesity treatment in Germany and Sweden: A multiyear, two-cohort observational study

**DOI:** 10.1371/journal.pmed.1004909

**Published:** 2026-07-09

**Authors:** Marie Auzanneau, Resthie R. Putri, Martin Wannack, Pernilla Danielsson, Stephanie Brandt-Heunemann, Claude Marcus, Susann Weihrauch-Blüher, Stefanie Lanzinger, Emilia Hagman

**Affiliations:** 1 Institute of Epidemiology and Medical Biometry, Ulm University, Ulm, Germany; 2 German Center for Diabetes Research (DZD), Neuherberg, Germany; 3 German Center for Child and Adolescent Health (DZKJ), Partner Site Ulm, Ulm, Germany; 4 Division of Pediatrics, Department of Clinical Science, Intervention and Technology, Karolinska Institutet, Stockholm, Sweden; 5 Department of Medical Epidemiology and Biostatistics, Karolinska Institutet, Stockholm, Sweden; 6 Department of Pediatric Endocrinology and Diabetology, Charité – Universitätsmedizin Berlin, Berlin, Germany; 7 Division of Paediatric Endocrinology and Diabetes, Department of Paediatrics and Adolescent Medicine, Ulm University Medical Center, Ulm, Germany; 8 Clinic for Pediatrics, University Medicine of Halle (Saale), Halle, Germany; London School of Hygiene and Tropical Medicine, UNITED KINGDOM OF GREAT BRITAIN AND NORTHERN IRELAND

## Abstract

**Background:**

Whether socioeconomic status (SES) influences obesity treatment response among children remains unclear. This study aimed to examine the association between SES and response to health behavior and lifestyle pediatric obesity treatment over 3 years in two European countries.

**Methods and findings:**

In this two-cohort study, data were obtained from the Swedish Childhood Obesity Treatment Register (BORIS) and the German/Austrian/Swiss Adiposity Patients Register (APV). SES was divided into quintiles, using individual-level indicators as a composite index in Sweden and an area-level index in Germany. Treatment response was assessed as change in body mass index standard deviation score (BMI SDS) using linear mixed-effects models, obesity remission using Cox regression, and treatment discontinuation within six months using mixed-effects logistic regression. Analyses were stratified by country and adjusted for sex, baseline obesity class, age group, and migration background.

Among 45,804 children with obesity who received health behavior and lifestyle obesity treatment, 31,293 (18,588 in Sweden and 12,705 in Germany) received at least six months of treatment. In both countries, higher baseline BMI SDS was associated with lower SES, <0.001. Associations between SES and change in BMI SDS differed between countries. In Sweden, SES was not associated with change in BMI SDS over time (*p* = 0.143). In Germany, higher SES was associated with greater reductions in BMI SDS (*p* < 0.001), with the largest decreases observed in the highest SES quintile after 2 years of treatment, −0.36 (95% CI [−0.38, −0.34]), sustained at 3 years (−0.34, 95% CI [−0.38, −0.29]). The difference in BMI SDS reduction between the highest and the lowest SES was greatest after 2 years, −0.12 (95% CI [−0.13, −0.12]) than after 3 years, −0.05 (95% CI [−0.07, −0.03]). Obesity remission showed a socioeconomic gradient in both countries, with higher adjusted probabilities in the highest versus lowest SES quintile (Sweden: 0.49 versus 0.37; Germany: 0.53 versus 0.45), corresponding to rate ratios of 1.63 (95% CI [1.44, 1.86]; *p* < 0.001) and 1.35 (95% CI [1.22, 1.50]; *p* < 0.001), respectively. Treatment discontinuation also differed by country. In Germany, higher SES compared to the lowest SES was associated with lower odds of discontinuation,OR of 0.82 (95% CI [0.74, 0.90]; *p* < 0.001), whereas no association was observed in Sweden. Residual confounding due to unavailable clinical and familial characteristics could not be ruled out.

**Conclusions:**

SES was associated with pediatric obesity treatment outcomes, but patterns differed between countries. While socioeconomic gradients in remission were observed in both settings, inequalities in BMI SDS reduction and treatment discontinuation were evident only in Germany, pointing to potential roles of both measurement differences and contextual factors.

## Author summary

**Why was this study done?:**

- Previous studies examining socioeconomic status (SES) and pediatric obesity treatment response have generally been small, single-center, and short-term, and have reported inconsistent results
- Systematic reviews have highlighted the lack of large-scale, multicenter, and long-term data, particularly from countries with universal healthcare systems, and have called for cross-national analyses to clarify how social determinants influence obesity treatment outcomes in children.

**What did the researchers do and find?:**

- This study included over 31,000 children and adolescents with obesity receiving health behavioral and lifestyle treatment in Sweden and Germany.
- Using harmonized socioeconomic indicators, we demonstrate that lower SES is associated with lower probability of obesity remission over 3 years in both countries. In addition, lower SES was associated with smaller body mass index standard deviation score (BMI SDS) reductions as well as higher rates of early treatment discontinuation in Germany.
- The magnitude of associations differed between countries, suggesting that methodological differences or national contexts, including welfare systems, may shape socioeconomic gradients in treatment outcomes.

**What do these findings mean?:**

- Socioeconomic disparities influence not only obesity prevalence and degree of obesity at treatment initiation, but may also affect response to obesity treatment.
- Our findings suggest that families with lower SES face barriers to optimal treatment response, i.e., obesity remission, even within structured healthcare systems. The study’s main limitation is that the findings may have been influenced by clinical or family-related factors that were not captured in the data.
- Studies evaluating mediating and modifiable factors that affect this association would be beneficial for both healthcare and for socioeconomically disadvantaged families.

## Introduction

The latest WHO European Childhood Obesity Surveillance Initiative revealed that 11% of 7- to 9-year-olds in the region have obesity [1]. Children and adolescents with obesity face a wide range of complications, including risk of cardiometabolic comorbidities, psychosocial challenges, and even premature mortality [2]. As favorable response to pediatric obesity treatment has substantial long-term health benefits [3], it is important to ensure equitable access to, and success in, obesity treatment.

The prevalence of obesity is unevenly distributed across pediatric populations. In high-income countries, children from lower socioeconomic status (SES) backgrounds have higher rates of obesity [4], which may be explained by a range of interrelated factors, including limited access to nutritious foods and increased exposure to obesogenic environments. Moreover, obesity is intertwined with broader socioeconomic disparities, including differences in educational attainment, across all SES groups [5].

Low family income has been associated with lower compliance to pediatric obesity treatment [6]. In one single-center study from the US, low income was associated with less weight loss after 4 months but not after 12 months [7]. In three other single-center studies from Europe, SES was not associated with treatment response after 2–3 years of treatment [8–10]. Despite these efforts, there remains a remarkable lack of high-quality, long-term, large-scale, and multicenter studies investigating the influence of SES on pediatric obesity treatment outcomes [11].

To address this gap, we analyzed cohorts from Sweden and Germany to assess whether associations between SES and treatment outcomes are consistent across different healthcare settings. A 3-year follow-up period was chosen to capture clinically meaningful treatment outcomes while minimizing loss to follow-up.

The primary study question was whether, and to what extent, SES is associated with the response to health behavior and lifestyle pediatric obesity treatment over 3 years in two high-income European countries. Secondary study questions were whether SES affects the likelihood of achieving obesity remission over the same time, as well as the likelihood of discontinuing treatment within the first six months. We hypothesize that SES is associated with the effectiveness of obesity treatment and non-adherence to such programs.

## Methods

### Study design and Participants

In this two-cohort study, data were obtained from the world’s two largest registries documenting pediatric obesity treatment, the Swedish Childhood Obesity Treatment Register (BORIS) and the German/Austrian/Swiss Adiposity Patients Register (APV) [12,13].

Patients in Sweden and Germany with obesity according to IOTF [14], who were between 3.0 and <17.0 years old at obesity treatment initiation (2000–2020) were eligible for inclusion. We excluded obesity associated syndromes, other genetic syndromes, insulin-dependent diabetes, treatment with systemic corticoids, Cushing’s disease, not well-controlled hypothyroidism, patients who received surgical or pharmacological obesity treatment, or those with missing data on SES. Additionally, to be included in the analyses of treatment response, patients had to remain in treatment for at least 6 months; otherwise, they were considered cases of early treatment discontinuation. The full procedure of inclusion and list for exclusion criteria is provided in Fig A and Table A in S1 File.

In both Sweden and Germany, first-line treatment for pediatric obesity consists of health behavioral and lifestyle therapy, targeting diet, physical activity, sedentary behavior, and broader behavior change such as daily routines. Treatment is sought to be individualized and may be delivered in a multidisciplinary, family-based or group-based format. During the period covered by the present study, GLP-1 receptor agonists were not yet approved for use in the pediatric population.

The study followed the Strengthening the Reporting of Observational Studies in Epidemiology (STROBE) reporting guidelines (S1 Checklist). The analysis plan was developed a priori (S2 File). In contrast to the initial analysis plan, data were analyzed separately by country, with results stratified by Sweden and Germany rather than pooled, as the outcomes differed between the countries. For the Swedish data, ethical approval was obtained by the regional Ethics Committee in Stockholm, Sweden for linking individuals in BORIS with national health and welfare registers (No. 2016/922–31/1, amendment 2020-02707) and sending pseudonymized data to Ulm University, Germany (No. 2020–05646). For the APV data, informed consent was obtained in participating centers and the Ethics Committee of Ulm University, Germany (No. 133/22) has approved the APV initiative as well as the collection and analysis of anonymized data for quality assurance and research.

### Data sources

BORIS and APV are prospective registries for the long-term monitoring of pediatric obesity treatment in Sweden and Germany, respectively [12,13]. BORIS has high coverage of pediatric obesity treatment (94% in 2023 and 92% in 2024), and APV covers the vast majority of the specialized treatment centers in Germany. However, both registers include only children who access obesity treatment and do not represent all children with obesity in the general population.

In Sweden, all residents are assigned a unique personal identification number, which was used to link BORIS to national registers by the governmental agencies Statistics Sweden and the National Board of Health and Welfare. Medical registers included the Prescribed Drug Register (all prescribed and dispensed medication), the Patient Register (diagnosis and surgical information from inpatient and outpatient specialist care), and the Cause of Death Register (date and cause of death). Data from medical registers, which are held by the National Board of Health and Welfare and are updated continuously, was used for the process of inclusion and exclusion. The Total Population Register, held by Statistics Sweden and updated annually, was used to obtained data on migration status. The Longitudinal Integrated Database for Health Insurance and Labour Market Studies register, also held by Statistics Sweden, was used to estimate SES; it contains demographic data, including income, highest achieved education, and occupational status on an individual level [15].

APV is a standardized multicenter database for centers providing outpatient or inpatient care for individuals with overweight or obesity, launched in Germany in 2000 and later also used in Austria and Switzerland [16]. In the present study, we have used data from Germany exclusively, because of their representativeness and the availability of nationwide socioeconomic indicators in this country (see below). In Germany, 236 hospitals, medical centers, and practices specialized in obesity treatment are currently using APV to document demographic, anthropometric, and metabolic characteristics of their patients. Twice yearly, the participating centers transmit their anonymized local data to Ulm University. After plausibility checks, correction, and validation, data are aggregated into the cumulative database [16]. For the present analysis, we excluded inpatient rehabilitation centers (*n* = 31), as this type of care has no equivalent in Sweden.

### Variables and definitions

#### Socioeconomic status.

SES, the main exposure, was categorized into quintiles based on distributions in the general population, with quintile 1 representing the lowest SES and quintile 5 the highest.

In Germany, SES was assessed using the German Index of Socioeconomic Deprivation (GISD 2019). Developed by the German Robert Koch Institute, the GISD encompassed aggregated data at municipality and district level (population size between 10 and 1 million habitants) in education (33.3%), occupation (33.3%), and income (33.3%) [17]. The GISD data is freely available for epidemiological research and can be downloaded from the following link: https://doi.org/10.5281/zenodo.6840304. The GISD has been proven to be a valuable tool to assess socioeconomic inequalities in health conditions and their contextual determinants in Germany [17,18]. The GISD score has been categorized into quintiles based on its distribution in the general population and quintiles were assigned to patients based on the zip code of their residential address. Children without GISD assignment due to missing or wrong postal code (*n* = 5,561 of 28,077, 19.8%), or residence outside Germany (*n* = 1,708 of 28,077, 6.1%), were excluded from the analysis, Fig A in S1 File (“available data on exposure”).

In Sweden, the composite SES variable was based on parental education, disposable income, and employment, and was constructed to reflect the structure of the GISD, although operationalized at the individual level. If a child was adopted, data of adoptive parents was used. Data of these components were obtained from The Longitudinal Integrated Database for Health Insurance and Labour Market Studies register [15] the year (or at the closest year) when the child initiated the obesity treatment. Educational attainment was categorized as compulsory school (score 1), upper secondary school (score 2), and college or university (score 3). Index-adjusted disposable income was divided into quintiles based on general population data derived from a reference population of parents of BORIS comparators matched on sex, year of birth, and residential district, and assigned scores from 1 (lowest) to 5 (highest). Employment status was scored as 1 (unemployed), 2 (student), or 3 (employed). Each component was weighed equally to mirror the GISD. Parental SES was calculated as the average of maternal and paternal SES scores. The resulting parental SES was then categorized into quintiles, based on its distribution in the reference population from the general population.

#### Response to pediatric obesity treatment and treatment discontinuation.

Treatment response, the primary outcome interest, was assessed using two measures: the body mass index standard deviation score (BMI SDS) for age and sex [14], and the cumulative change of BMI SDS from baseline. Data was retrieved from BORIS and APV.

As secondary outcomes, obesity remission and treatment discontinuation within the first 6 months were assessed. Obesity remission was defined as no longer meeting the criteria for obesity, according to the International Obesity Task Force [14] at the last recorded visit within 3 years of treatment initiation. Treatment discontinuation was defined as receiving less than 6 months of treatment.

#### Degree of obesity.

Degree of obesity as a covariate was categorized into obesity class 1, 2, or 3 based on the baseline BMI SDS corresponding to adult BMI thresholds of 30, 35, and 40 kg/m^2^, respectively [14,19]. Data was retrieved from BORIS and APV.

#### Age categories.

Age at baseline was categorized into three age groups; 3–9.9 years, 10–13.9 years and 14–16.9 years. Data was retrieved from BORIS and APV.

#### Migration background.

Migration background was included to capture sociocultural and structural factors, such as differences in language, cultural context, and familiarity with the healthcare system, which may influence treatment access and outcomes. Individuals in BORIS were categorized as Nordic (born in, and with 1 or 2 parents born in, Sweden, Norway, Denmark, Finland, or Iceland) or non-Nordic. For individuals in APV, migration background was defined as children or at least 1 parent being born outside Germany. Children without documentation of migration background were assumed to have no migration background. Data was retrieved from Statistics Sweden and APV.

#### Sex.

Sex was dichotomized. In Sweden, data was retrieved from Statistics Sweden, and in Germany data was retrieved from APV.

### Statistical analyses

All statistical analyses were performed for each country separately. Descriptive analyses were reported as proportions for categorical variables, and as median, quartile 1 (Q1), and quartile 3 (Q3) for continuous variables. Outcomes were compared between subgroups using Wilcoxon rank sum test for continuous variables or Chi^2^-test for binary variables. *P* values were adjusted for multiple testing according to the Bonferroni–Holm method. To investigate the association between SES quintiles and the response to pediatric obesity treatment, we carried out longitudinal analyses taking into account every BMI SDS measurement from the initial visit up to 3 years of treatment for each patient. Mean BMI SDS and mean cumulative change in BMI SDS were estimated across SES quintiles and time (in months rounded) using linear mixed-effects models with spline. These models accounted for repeated measurements. The models were adjusted for sex, baseline age group, and migration background. Additionally, baseline obesity class was included in the adjustment when cumulative change in BMI SDS was the outcome, as the initial degree of obesity may differ across socioeconomic groups and potentially influence subsequent change. In light of ongoing methodological debate regarding baseline adjustment and change-score analyses [20], and their differing underlying assumptions, sensitivity analyses without adjustment for baseline obesity class, and with BMI SDS instead of obesity class were performed. There were no missing data for the variables included in the analyses.

Probabilities and rates ratios of obesity remission over 3 years by SES quintiles were calculated using a Cox regression model adjusted for sex, age group, obesity class at baseline, and migration background.

To calculate the probabilities and odds ratios of treatment discontinuation by SES quintiles, a mixed-effect logistic regression model adjusted for sex, age group, obesity class at baseline, and migration background was performed. Coefficients, odds ratios (OR), rates/hazard ratios from regression models are reported with their 95% confidence intervals (95% CI) and *p*-values. SES quintile 1 (Q1) was used as the reference group. Statistical significance was defined as a p-value of <0.05.

Data management in Sweden was carried out using SAS version 9.4 (SAS Institute) and Stata version 16 (StataCorp). Data management for APV and statistical analysis for both cohorts (BORIS and APV) were carried out in Ulm, Germany, using SAS version 9.4 (SAS Institute), build TS1M7 on a Window Server mainframe.

## Use of artificial intelligence

Artificial intelligence (AI) tools were not used for study design, data analysis, interpretation of results, literature searching, reference selection, or any other intellectual contribution. All scientific content, analyses, interpretations, and conclusions were developed and verified by the authors.

## Results

### Baseline characteristics by SES

Of 45,804 children and adolescents with obesity, a total of 31,293 (49.4% female) had received at least six months of obesity treatment and were included in the analyses examining the association between SES and treatment response. This analytic sample comprised 18,588 patients from Sweden (46.7% female) with a median follow-up time of 23.7 (Q1,Q3 [13.7, 30.6]) months and 12,705 from Germany (53.2% female) with a median follow-up time of 14.6 (Q1,Q3 [10.9, 24.6]) months. At treatment initiation, the median age was 9.7 (Q1,Q3 [7.3, 12.3]) years in Sweden and 11.2 (Q1,Q3 [9.3, 13.0]) years in Germany. The median BMI SDS was 2.82 (Q1,Q3 [2.55, 3.16]) in Sweden and 2.77 (Q1,Q3 [2.51, 3.07]) in Germany. Lower SES was more common than higher SES in the study population in both countries. Moreover, BMI SDS at treatment initiation was higher among those with lower SES, p for both countries <0.001. A key difference between the national cohorts was that, in Sweden, the proportion of individuals with a migration background was higher in the lowest SES compared to the highest SES (55.3% in SES Q1 versus 14.5% in SES Q5, *p* < 0.001); conversely, in Germany, the opposite pattern was observed (14.9% in SES Q1 versus 23.3% in SES Q5, *p* < 0.001). Characteristics of the study population are provided in Table 1.

**Table 1:** Characteristics of included patients by SES quintile, stratified by country.

	SES quintile 1^1^	SES quintile 2	SES quintile 3	SES quintile 4	SES quintile 5	*p*-value^2^	Total
**Germany**	*n* = 3 533	*n* = 2 392	*n* = 2 570	*n* = 2 298	*n* = 1 912		*n* = 12 705
Female sex, *n* (%)	1,903 (53.9)	1,274 (53.3)	1,335 (51.9)	1,221 (53.1)	1,032 (54.0)	0.508	6,765 (53.2)
Migrant background, *n* (%)	527 (14.9)	362 (15.1)	545 (21.2)	535 (23.3)	446 (23.3)	<0.001	2,415 (19.0)
Age at treatment initiation, median (Q1, Q3)	11.2 (9.1, 13.1)	10.9 (8.8, 12.9)	11.2 (9.3, 12.9)	11.4 (9.6, 13.1)	11.5 (9.7, 13.1)	<0.001	11.2 (9.3, 13.0)
3 to <10 years, *n* (%)	1,240(35.1)	895 (37.4)	836 (32.5)	668 (29.1)	550 (28.8)	<0.001	4,189 (33.0)
10 to <14 years, *n* (%)	1,781 (50.4)	1,161 (48.5)	1,382 (53.8)	1,282 (55.8)	1,113 (58.2)	<0.001	6,719 (52.9)
14 to <17 years, *n* (%)	512 (14.5)	336 (14.0)	352 (13.7)	348 (15.1)	249 (13.0)	0.324	1,797 (14.1)
BMI SDS at treatment initiation, median (Q1, Q3)	2.82 (2.55, 3.13)	2.79 (2.52, 3.10)	2.78 (2.51, 3.07)	2.75 (2.50, 3.05)	2.74 (2.49, 3.02)	<0.001	2.77 (2.51, 3.07)
Obesity class I, *n* (%)	1,970 (55.8)	1,402 (58.6)	1,536 (59.8)	1,435 (62.4)	1,214 (63.5)	<0.001	7,557 (59.5)
Obesity class II, *n* (%)	1,067 (30.2)	687 (28.7)	750 (29.2)	611 (26.6)	528 (27.6)	0.036	3,643 (28.7)
Obesity class III, *n* (%)	496 (14.0)	303 (12.7)	284 (11.1)	252 (11.0)	170 (8.9)	<0.001	1,505 (11.8)
BMI at treatment initiation, median (Q1, Q3)	29.0 (26.2, 32.4)	28.5 (25.5, 31.6)	28.7 (26.1, 31.8)	29.0 (26.4, 32.0)	29.0 (26.5, 31.7)	<0.001	28.8 (26.1, 31.9)
Treatment duration (months), median (Q1, Q3)	15.7 (10.8, 25.1)	13.2 (10.1, 23.6)	15.5 (11.1, 25.9)	13.6 (10.8, 22.7)	13.5 (11.0, 23.0)	<0.001	14.6 (10.9, 24.6)
**Sweden**	*n* = 6 726	*n* = 7 143	*n* = 1 527	*n* = 2 349	*n* = 843		*n* = 18 588
Female sex, *n* (%)	3,098 (46.1)	3,361 (47.1)	709 (46.4)	1,099 (46.8)	416 (49.3)	0.843	8,683 (46.7)
Migrant background, *n* (%)	3,717 (55.3)	2,137 (29.9)	308 (20.2)	443 (18.9)	122 (14.5)	<0.001	6,727 (36.2)
Age at treatment initiation, median (Q1, Q3)	9.8 (7.2, 12.4)	9.6 (7.2, 12.3)	9.6 (7.3, 12.2)	9.8 (7.5, 12.4)	9.7 (7.5, 12.2)	0.539	9.7 (7.3, 12.3)
3 to <10 years, *n* (%)	2,819 (41.9)	3,177 (44.5)	670 (43.9)	985 (41.9)	356 (42.2)	0.023	8,007 (43.1)
10 to <14 years, *n* (%)	1,988 (29.6)	1,995 (27.9)	448 (29.3)	708 (30.1)	263 (31.2)	0.075	5,402 (29.1)
14 to <17 years, *n* (%)	1,919 (28.5)	1,971 (27.6)	409 (26.8)	656 (27.9)	224 (26.6)	0.502	5,179 (27.9)
BMI SDS at treatment initiation, median (Q1, Q3)	2.91 (2.61, 3.27)	2.83 (2.56, 3.16)	2.74 (2.50, 3.08)	2.70 (2.49, 3.00)	2.62 (2.45, 2.89)	<0.001	2.82 (2.55, 3.16)
Obesity class I, *n* (%)	3,231 (48.0)	3,936 (55.1)	935 (61.2)	1,561 (66.5)	617 (73.2)	<0.001	10,280 (55.3)
Obesity class II, *n* (%)	1,978 (29.4)	2021 (28.3)	384 (25.1)	552 (23.5)	162 (19.2)	<0.001	5,097 (27.4)
Obesity class III, *n* (%)	1,517 (22.6)	1,186 (16.6)	208 (13.6)	236 (10.0)	64 (7.6)	<0.001	3,211 (17.3)
BMI at treatment initiation, median (Q1, Q3)	27.3 (23.9, 31.1)	26.5 (23.4, 30.3)	26.1 (23.2, 29.5)	26.2 (23.1, 29.4)	25.6 (22.9, 29.1)	<0.001	26.6 (23.5, 30.4)
Treatment duration (months), median (Q1, Q3)	24.4 (14.2, 31.1)	24.1 (13.8, 30.9)	22.4 (13.5, 29.9)	21.0 (13.1, 29.0)	20.2 (12.5, 28.3)	<0.001	23.7 (13.7, 30.6)

BMI SDS, body mass index standard deviation score; *n*, number; Q1, quartile 1; Q3, quartile 3; SES, socioeconomic status.

^1^SES quintile 1 represents the lowest SES category, whereas SES quintile 5 represents the highest SES category.

^2^*P*-values for comparison by SES quintile are derived from Wilcoxon rank sum test for continuous variables and Chi^2^-test for binary variables, with adjustment for multiple testing according to the Bonferroni–Holm method.

### The association between SES and response to obesity treatment

**Fig 1:**
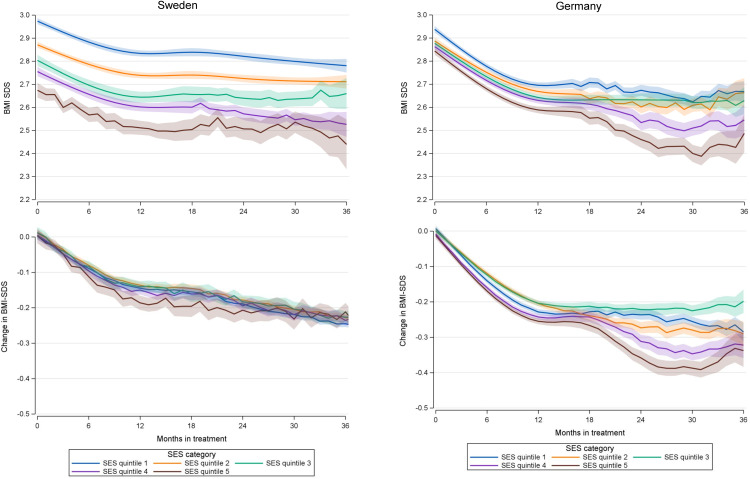
Absolute BMI SDS (top panel) and change in BMI SDS from baseline (bottom panel) over 3 years of treatment, shown separately for Sweden and Germany, by SES quintile (Q1–Q5), where Q1 represents the least advantaged and Q5 the most advantaged. Estimates are derived from linear mixed-effect models with spline, including repeated measurements and country as random intercept, and adjusted for sex, age group, migration background, obesity class at baseline (except for BMI SDS as outcome). BMI SDS, body mass index standard deviation score; Q, quintile; SES, socioeconomic status.

**Fig 2:**
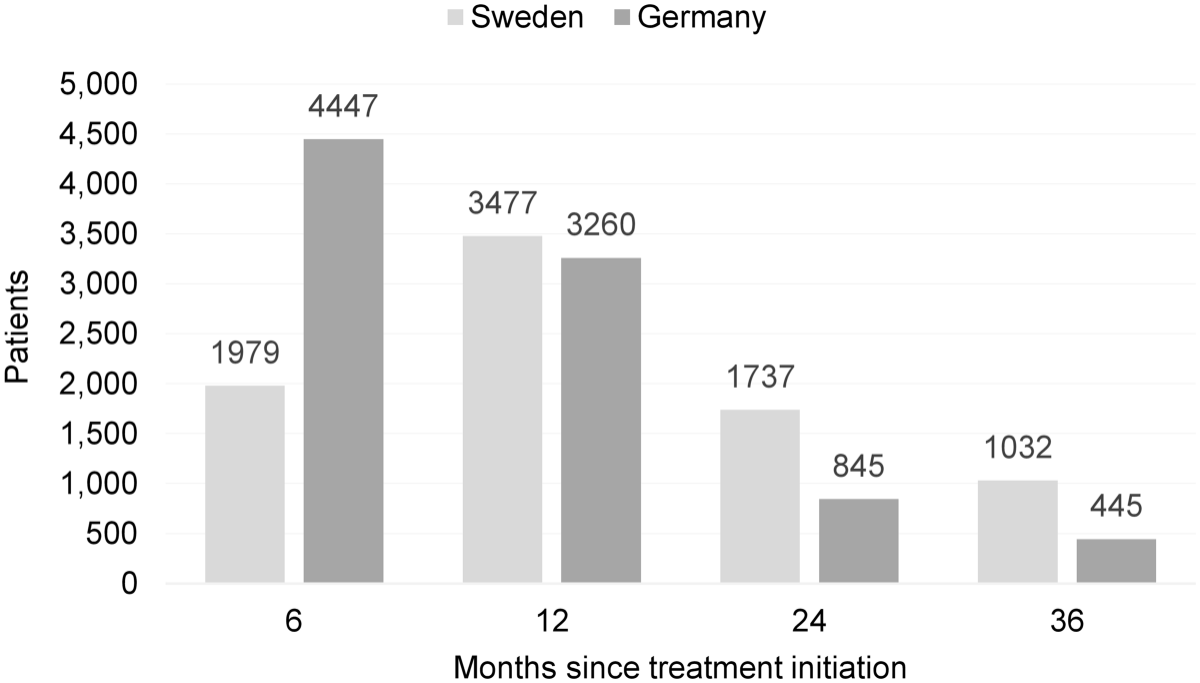
Number of patients contributing with follow-up data at different timepoints since treatment initiation.

In both countries, a lower SES was associated with a higher BMI SDS at treatment initiation. However, the association between SES and change in BMI SDS over time showed different patterns in Sweden and Germany, Fig 1. In Sweden, there was no evidence that SES modified the change in BMI SDS over time, as the SES-by-time interaction was not statistically significant (*p* = 0.143). However, in Germany, an association between SES and response to pediatric obesity treatment was observed (interaction term *p* < 0.001), where patients in the highest SES quintile (Q5) demonstrated the greatest reductions in BMI SDS, and patients in middle SES (Q3) had smallest reductions. Adjusted mean changes in SES Q5 were –0.25 (95% CI [−0.26, −0.25]) after 1 year, −0.36 (95% CI [−0.38, −0.34]) after 2 years, and −0.34 (95% CI [−0.38, −0.29]) after 3 years of treatment. In contrast, patients from SES Q3 showed smallest reductions: −0.20 (95% CI [−0.21, −0.20]) after 1 year and −0.22 (95% CI [−0.24, −0.21]) after 2 years, and −0.20 (95% CI [−0.23, −0.17]) after 3 years. Compared to the lowest SES quintile (Q1), BMI SDS reduction was significantly greater in the highest SES quintile (Q5). The difference in difference between SES Q5 and SES Q1 was greatest after 2 years: −0.12 (95% CI [−0.13, −0.12]), and less pronounced after 3 years: −0.05 (95% CI [−0.07 to −0.03]). Due to differences in baseline obesity severity, absolute BMI SDS at 36 months remained higher in children from lower SES backgrounds (Fig 1). Unadjusted results and sensitivity analyses with BMI SDS instead of obesity class, and without obesity class adjustment were similar to adjusted as shown in Figs B, C, and D in S1 File. Number of patients contributing with follow-up data at different timepoints is presented in Fig 2.

### Probability of obesity remission

During the study period, 4,217 (22.7%) patients in Sweden and 3,766 (29.6%) in Germany experienced obesity remission during treatment. In both countries, SES had a significant effect on the probability of obesity remission, even after adjustment for confounders (*p* < 0.001 in Sweden and in Germany). The adjusted probability of remission after up to 3 years of treatment was lowest in the least socioeconomically advantaged quintile, Q1 (0.37 (95% CI [0.35–0.38]) in Sweden and 0.45 (95% CI [0.43–0.48]) in Germany and increased progressively with higher SES, reaching 0.49 (95% CI [0.46, 0.52]) in Sweden and 0.53 (95% CI [0.50, 0.55]) in Germany in the highest SES quintile (Q5), indicating a dose–response relationship. Correspondingly, the adjusted rate ratio for obesity remission in Q5 compared to Q1 was 1.63 (95% CI [1.44, 1.86]; *p* < 0.001) in Sweden and 1.35 (95% CI [1.22, 1.50]; *p* < 0.001) in Germany, Table 2. Probabilities of remission over the course of treatment are provided in Fig E in S1 File.

**Table 2:** Adjusted probabilities and rate ratios (95% CI) of obesity remission over 3 years of treatment, by country^1^.

	Germany	Sweden	Germany	Sweden
	Unadjusted probability (95% CI)		Adjusted probability (95% CI)	
SES Q1 (lowest)	0.45 (0.42, 0.48)	0.33 (0.31, 0.35)	0.45 (0.43, 0.48)	0.37 (0.35, 0.38)
SES Q2	0.51 (0.47, 0.54)	0.38 (0.36, 0.40)	0.49 (0.46, 0.51)	0.37 (0.35, 0.39)
SES Q3	0.52 (0.48, 0.55)	0.46 (0.43, 0.50)	0.49 (0.46, 0.51)	0.42 (0.39, 0.44)
SES Q4	0.56 (0.53, 0.60)	0.51 (0.48, 0.54)	0.52 (0.49, 0.55)	0.43 (0.41, 0.45)
SES Q5 (highest)	0.59 (0.55, 0.62)	0.63 (0.59, 0.67)	0.53 (0.50, 0.55)	0.49 (0.46, 0.52)
	Unadjusted rate ratio (95% CI); *p*		Adjusted rate ratio (95% CI); *p*	
SES Q1 (lowest)	Ref	Ref	Ref	Ref
SES Q2	1.18 (1.07, 1.31); 0.0008	1.19 (1.10, 1.28); *p* < 0.0001	1.14 (1.04, 1.26); *p* = 0.0081	1.02 (0.95, 1.10); *p* = 0.558
SES Q3	1.22 (1.11, 1.35); *p* < 0.0001	1.56 (1.39, 1.74); *p* < 0.0001	1.15 (1.05, 1.27); *p* = 0.0042	1.22 (1.09, 1.37); *p* = 0.0006
SES Q4	1.39 (1.26, 1.53); *p* < 0.0001	1.78 (1.62, 1.95); *p* < 0.0001	1.31 (1.19, 1.45); *p* < 0.0001	1.29 (1.17, 1.43); *p* < 0.0001
SES Q5 (highest)	1.47 (1.33, 1.36); *p* < 0.0001	2.47 (2.18, 2.78); *p* < 0.0001	1.35 (1.22, 1.50); *p* < 0.0001	1.63 (1.44, 1.86); *p* < 0.0001

CI, confidence interval; Q, quintile; Ref, reference; SES, socioeconomic status.

^1^Cox-regression adjusted for sex, age group, obesity class, and migration background.

### Probability of treatment discontinuation

In the sample of 45,804 patients, 6,399 (25.6%) patients in Sweden and 8,094 (38.9%) in Germany did not remain in treatment after 6 months. In Germany, those with higher SES (Q5) had a lower risk of treatment discontinuation compared to the lowest SES quintile (Q1), adjusted OR for SES Q5 versus Q1: 0.82 (95% CI [0.74, 0.90]; *p* < 0.001). However, the absolute difference in the probability of not remaining in treatment after 6 months was modest. In Sweden, treatment discontinuation did not differ significantly between SES categories. Probabilities and ORs of treatment discontinuation are provided in Table 3.

**Table 3:** Adjusted probabilities and odds ratios (95% CI) of treatment discontinuation within 6 months, by country^1^.

	Germany	Sweden	Germany	Sweden
	Unadjusted probability (95% CI)		Adjusted probability (95% CI)	
SES Q1 (lowest)	0.38 (0.37, 0.40)	0.24 (0.23, 0.25)	0.38 (0.37, 0.39)	0.23 (0.22, 0.24)
SES Q2	0.40 (0.38, 0.41)	0.23 (0.23, 0.24)	0.39 (0.38, 0.41)	0.24 (0.23, 0.24)
SES Q3	0.38 (0.37, 0.40)	0.24 (0.22, 0.25)	0.38 (0.37, 0.40)	0.24 (0.22, 0.26)
SES Q4	0.33 (0.32, 0.35)	0.22 (0.21, 0.24)	0.33 (0.32, 0.35)	0.23 (0.21, 0.24)
SES Q5 (highest)	0.33 (0.31, 0.35)	0.24 (0.22, 0.27)	0.33 (0.32, 0.35)	0.25 (0.22, 0.27)
	Unadjusted odds ratio (95% CI); p		Adjusted odds ratio (95% CI); p	
SES Q1 (lowest)	Ref	Ref	Ref	Ref
SES Q2	1.06 (0.98, 1.15); p= = 0.165	0.99 (0.92, 1.05); p= = 0.663	1.05 (0.97, 1.14); p= = 0.239	1.02 (0.95, 1.10); p= = 0.549
SES Q3	1.01 (0.93, 1.09); p= = 0.881	1.00 (0.89, 1.11); p= = 0.936	1.01 (0.93, 1.09); p= = 0.849	1.05 (0.93, 1.18); p= = 0.422
SES Q4	0.81 (0.74, 0.88); p< < 0.0001	0.92 (0.84, 1.02); p= = 0.100	0.82 (0.75, 0.89); p< < 0.0001	0.96 (0.87, 1.06); p= = 0.449
SES Q5 (highest)	0.80 (0.73, 0.88); p< < 0.0001	1.04 (0.902, 1.20); p= = 0.582	0.82 (0.74, 0.90); p< < 0.0001	1.09 (0.94, 1.26); p= = 0.236

CI, confidence interval; Q, quintile; Ref, reference; SES, socioeconomic status.

^1^Probabilities and odds ratios of treatment discontinuation by SES quintiles, are derived from a mixed-effect logistic regression model adjusted for sex, age group, obesity class at baseline, and migration background.

## Discussion

In this large two-country cohort study, SES was associated with several pediatric obesity treatment outcomes. Children with obesity from higher SES families demonstrated lower BMI SDS at baseline, and were more likely to experience obesity remission over 3 years in both Sweden and Germany. Additionally, in Germany, lower SES was associated with greater reductions in BMI SDS over 3 years, and with a higher likelihood of treatment discontinuation within 6 months, although to a modest extent. Although the associations with BMI SDS reduction and treatment discontinuation were more evident in Germany than in Sweden, comparisons between the two countries should be interpreted with caution.

Previous studies on SES and pediatric obesity treatment have reported inconsistent results, often constrained by small samples, single-center settings, and limited follow-up [6–11]. Our results extend this literature by quantifying the magnitude of socioeconomic differences in treatment response. In Germany, the difference in BMI SDS reduction between the highest and lowest SES quintiles reached 0.12 units after 2 years of treatment. Although modest in absolute terms, this difference may be clinically relevant, particularly as children from lower SES backgrounds presented with higher baseline BMI SDS. Consequently, even similar relative reductions may translate into persistently higher absolute BMI SDS which may contribute to differences in clinically relevant outcomes, such as remission, although the association between SES and remission persisted after adjustment for baseline obesity severity. These findings also suggest that earlier identification and treatment initiation among children from lower SES backgrounds could be important to improve long-term outcomes.

The probability of achieving remission showed a clear SES gradient, with higher SES associated with greater likelihood of remission across both countries. This gradient was particularly strong in Sweden, where remission rates for the highest SES quintile were more than double those of the lowest SES quintile. Unlike the continuous measure of BMI SDS, remission represents a categorical threshold, and our findings indicate that children from low SES families not only have more severe obesity at baseline but also face greater difficulty in experiencing obesity remission independently of baseline obesity severity. These results are in line with previous work from the US, which demonstrated that socioeconomically disadvantaged children were less likely to experience obesity remission [21].

Mediators of the association between SES and treatment response are not fully understood, but likely involve several, interrelated pathways. Limited economic resources may restrict access to healthy foods, organized physical activity, and transportation to healthcare visits [22]. Parental shift work or multiple jobs may limit attendance at appointments and support for lifestyle changes at home. SES-related residential differences may influence treatment outcomes via disparities in healthcare access and quality [23]. Moreover, limited health literacy, difficulties in navigating healthcare systems, and broader psychosocial stressors (e.g., financial strain, stigma, and reduced social support) may contribute [24–26]. Additionally, recent genomic work suggests that socioeconomic gradients in health, including obesity, may partly reflect intertwined environmental and heritable factors [27]. Future research should elucidate which mediators are modifiable, enabling targeted interventions and more equitable care.

The differences in association magnitude between the two countries may reflect both methodological and contextual factors. SES was assessed using individual-level indicators in Sweden and an area-level index in Germany, which capture different dimensions of socioeconomic position. Both family-level and area-level socioeconomic conditions have been shown to independently influence childhood obesity risk [28,29], suggesting that these measures are not directly interchangeable. Therefore, direct comparability between countries should be interpreted with caution. One possible explanation for the observed association between SES and change in BMI SDS in Germany, but not in Sweden, is that area-level conditions may play a greater role in treatment outcomes than individual-level SES. Additionally, the degree of obesity at baseline varied more across SES groups in Sweden than in Germany. Such differences in baseline characteristics may partly account for why BMI SDS reduction appeared stronger in one country than the other. Structural factors may also contribute. The absence of association between SES and BMI SDS reduction in Sweden compared to Germany could reflect the universal healthcare system, subsidized school meals, and other social support mechanisms that reduce barriers for families with lower SES. Although all children received health behavior and lifestyle obesity treatment, there may have been heterogeneity in how the treatment was delivered across the countries. Taken together, these factors may partly account for the observed differences between countries.

Despite this study’s large sample size, longitudinal follow-up, and the use of high-quality registry data from two countries, limitations should also be acknowledged. First, residual confounding cannot be excluded. Unmeasured factors such as parental BMI, health literacy, and family engagement may be associated with both SES and treatment outcomes. If more prevalent among higher SES groups, this could lead to overestimation of the observed associations. Another potential residual confounder is treatment intensity, as higher visit frequency has been shown to be associated with improved long-term treatment response [30]. Second, restricting analyses to individuals remaining in treatment for at least six months may have introduced selection bias. As early discontinuation was more common among lower SES groups (in Germany), the analytic sample may represent a more selected subgroup, potentially affecting SES differences over time. Third, in the absence of an untreated comparison group, we cannot distinguish treatment effects from the natural course of BMI development across different SES strata, limiting causal inference. Fourth, we were not able to stratify for subgroups, e.g., degree of obesity, as we deemed assuming representative sample size from month 0 to month 36 for each subgroup would be improbable. Finally, clustering at the treatment center level was not accounted for due to the large number of contributing centers (98 in Sweden and 159 in Germany) and patient transfers between them. While this may have led to slight underestimation of confidence intervals, the large number of centers also supports the generalizability of the findings.

In conclusion, this study demonstrates that socioeconomic disparities are associated with several pediatric obesity treatment outcomes in Germany, including lower rates of weight reduction and remission and higher rates of treatment discontinuation, and with the likelihood of obesity remission in Sweden. Although change in BMI SDS showed different patterns between the countries, absolute BMI SDS remained higher for socioeconomically disadvantaged groups after 3 years of treatment. Together, these findings emphasize the need for context-specific strategies to reduce socioeconomic inequalities in pediatric obesity treatment.

## Supporting information

S1 File Supplementary materials. **Fig A.** Flowchart APV (Germany) and BORIS (Sweden), respectively. **Table A.** Identification of exclusion criteria. **Fig B.** Unadjusted absolute BMI SDS and cumulative change in BMI SDS over 3 years of treatment by SES category. **Fig C.** Sensitivity analyses, with baseline BMI SDS adjustment instead of obesity class, of change in BMI SDS from baseline over 3 years of treatment. **Fig D.** Sensitivity analyses, without baseline obesity class adjustment of change in BMI SDS from baseline over 3 years of treatment. **Fig E.** Probability of obesity remission over 3 years by SES quintiles.
(DOCX)

S1 Checklist STROBE checklist. This checklist is licensed under a CCBY 4.0 license (https://creativecommons.org/licenses/by/4.0/deed.en). https://www.strobe-statement.org/checklists/.
(PDF)

S2 File Prospective analysis plan. (PDF)

S3 File Dataset used to generate figures of main findings. (ZIP)

## Abbreviations

AI: artificial intelligence
APV: German/Austrian/Swiss Adiposity Patients Register
BMI SDS: body mass index standard deviation score
BORIS: Swedish Childhood Obesity Treatment Register
GISD: German Index of Socioeconomic Deprivation
OR: odds ratios
SES: socioeconomic status
STROBE: Strengthening the Reporting of Observational Studies in Epidemiology
