# Supplementary material for: The association of socioeconomic status and response to pediatric health behavior and lifestyle obesity treatment in Germany and Sweden: A multiyear, two-cohort observational study
Source: PLoS Med. 2026 Jul 9;23(7):e1004909. doi: 10.1371/journal.pmed.1004909 (PMC13367900; doi:10.1371/journal.pmed.1004909)
Supplement: S1 File — Fig A. Flowchart APV (Germany) and BORIS (Sweden), respectively. Table A. Identification of exclusion criteria. Fig B. Unadjusted absolute BMI SDS and cumulative change in BMI SDS over 3 years of treatment by SES category. Fig C. Sensitivity analyses, with baseline BMI SDS adjustment instead of obesity class, of change in BMI SDS from baseline over 3 years of treatment. Fig D. Sensitivity analyses, without baseline obesity class adjustment of change in BMI SDS from baseline over 3 years of treatment. Fig E. Probability of obesity remission over 3 years by SES quintiles. (DOCX) [file pmed.1004909.s001.docx]

S1 File for

**The association of socioeconomic status and response to pediatric health behavior and lifestyle obesity treatment in Germany and Sweden: A multiyear, two-cohort observational study**

Marie Auzanneau, Resthie R Putri, Martin Wannack, Pernilla Danielsson, Stephanie Brandt-Heunemann, Claude Marcus, Susann Weihrauch-Blüher, Stefanie Lanzinger, Emilia Hagman

**Table of Content**

[**Fig A. Flowchart APV (Germany) and BORIS (Sweden) respectively** 2](#_Toc233704894)

[**Table A. Identification of exclusion criteria** 3](#_Toc233704895)

[**Fig B. Unadjusted absolute BMI SDS (top panel) and cumulative change in BMI SDS (bottom panel) over 3 years of treatment by SES category** 4](#_Toc233704896)

[**Fig C. Sensitivity analyses, with baseline BMI SDS adjustment instead of obesity class, of change in BMI SDS from baseline over three years of treatment** 5](#_Toc233704897)

[**Fig D. Sensitivity analyses, without baseline obesity class adjustment of change in BMI SDS from baseline over three years of treatment** 6](#_Toc233704898)

[**Fig E. Probability of obesity remission over three years by SES quintiles** 7](#_Toc233704899)

# **Fig A. Flowchart APV (Germany) and BORIS (Sweden) respectively**

* As our follow-up data end by the end of 2020, all individuals entering obesity treatment the same year was excluded.

| **Table A. Identification of exclusion criteria** | | |
| --- | --- | --- |
|  | Germany^1^ | Sweden^2^ |
| Genetic syndromes | Documentation of obesity associated syndromes | ICD-10 diagnosis code in the National Patient Register:  Mb Down (Q90), Prader Willi (Q871), Laurence Moon Bardet Biedl (Q878), Russel (Q871G), Noonan (Q871E), Klinefelter (Q98), Fragile-X (Q992)and Turner (Q96) |
| Craniopharyngioma | Documentation of obesity associated with endocrine diseases/disorders | ICD-10 codes for craniopharyngioma: D353, D444, and C752 |
| Insulin-dependent diabetes | Insulin documented as treatment | At least two diagnoses of T1D (ICD-10 E10) + at least two dispensed prescriptions of insulins (ATC code A10A) during obesity treatment. |
| Cushing’s disease | Documentation of obesity associated with endocrine diseases/disorders | Identified though ICD-10 code E24 |
| Systemic corticoid treatment | Documentation of systemic glucocorticoid treatment | Prescribed and dispensed ATC code H02 during obesity treatment |
| Metabolic bariatric surgery | Documentation of surgical intervention for obesity | Bariatric surgery during pediatric obesity treatment was defined by the combination of surgical procedure ‘JDF’ (Volume-restricting surgery on the ventricle) and diagnosis of obesity (ICD-10 code E66) |
| Pharmacological obesity treatment | Documentation of any pharmacological obesity treatment (including: orlistat, metformin, GLP1-RA) | Pharmacological obesity treatment during pediatric obesity treatment was identified from ATC codes A08A (anti-obesity pharmaceuticals) and A10BJ (GLP1-R analogs) |
| ^1^ Diagnoses and treatments based on physician’s documentation in the APV register  ^2^ Diagnoses based on ICD-10 codes and surgical procedures were obtained from the National Patient Register and pharmacological treatments were obtained from the National Prescribed Drug register using designated ATC codes. | | |

**Fig B. Unadjusted absolute BMI SDS (top panel) and cumulative change in BMI SDS (bottom panel) over 3 years of treatment by SES category.**

Sweden Germany


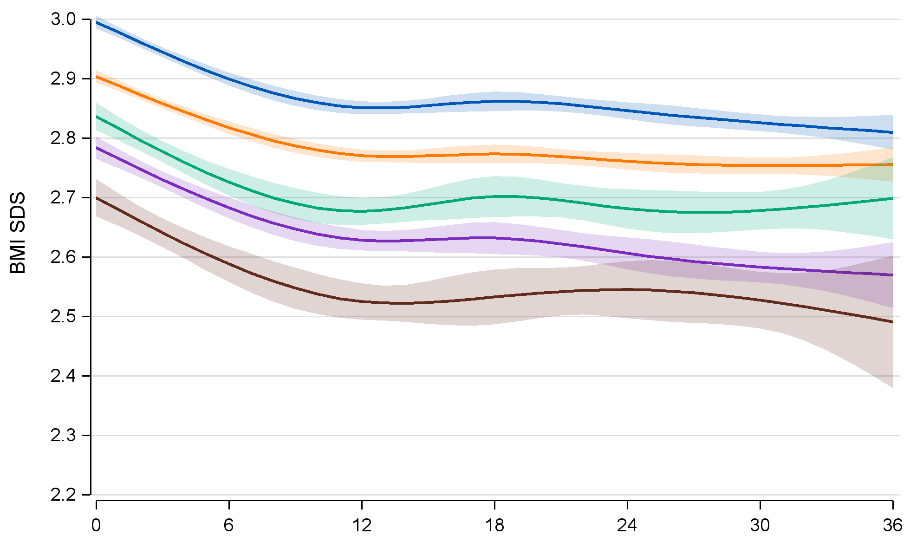

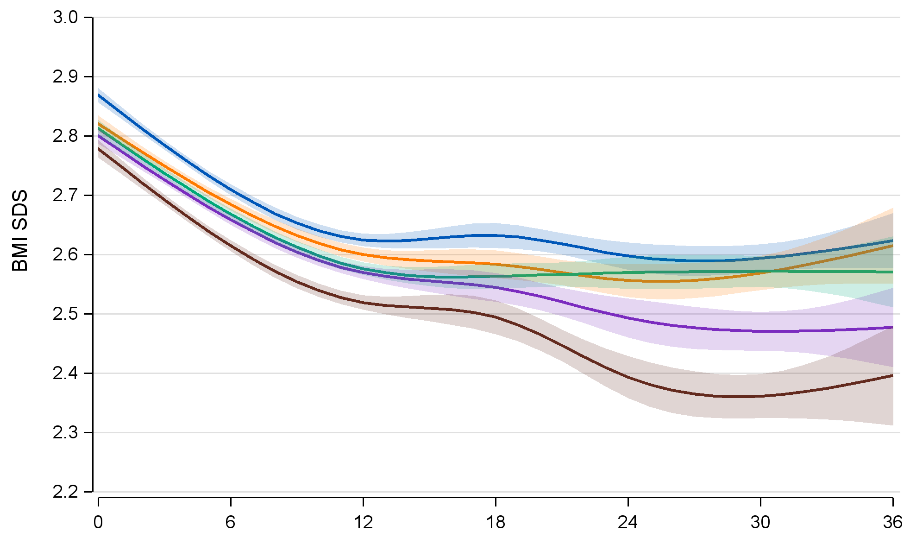


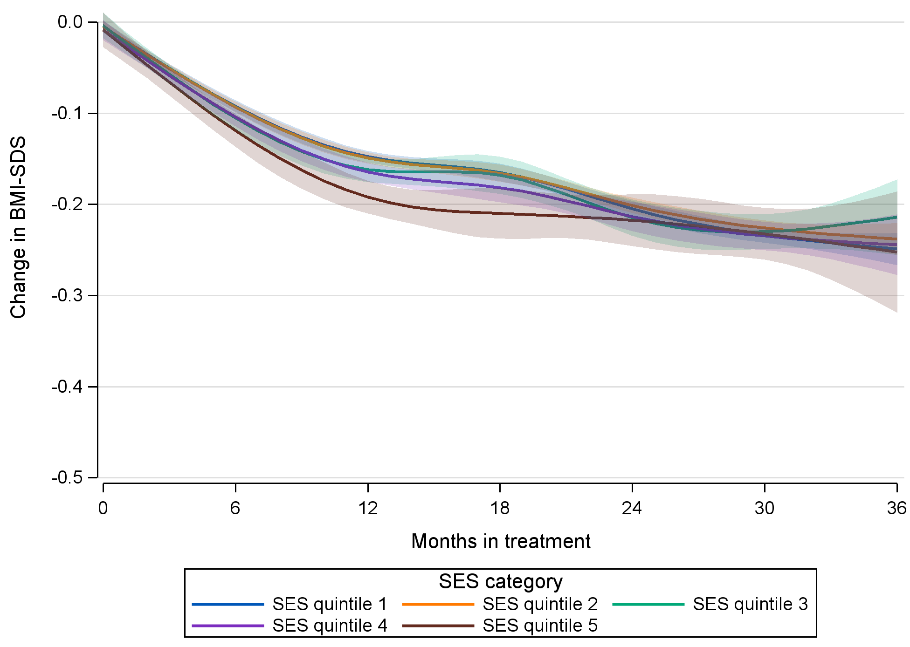

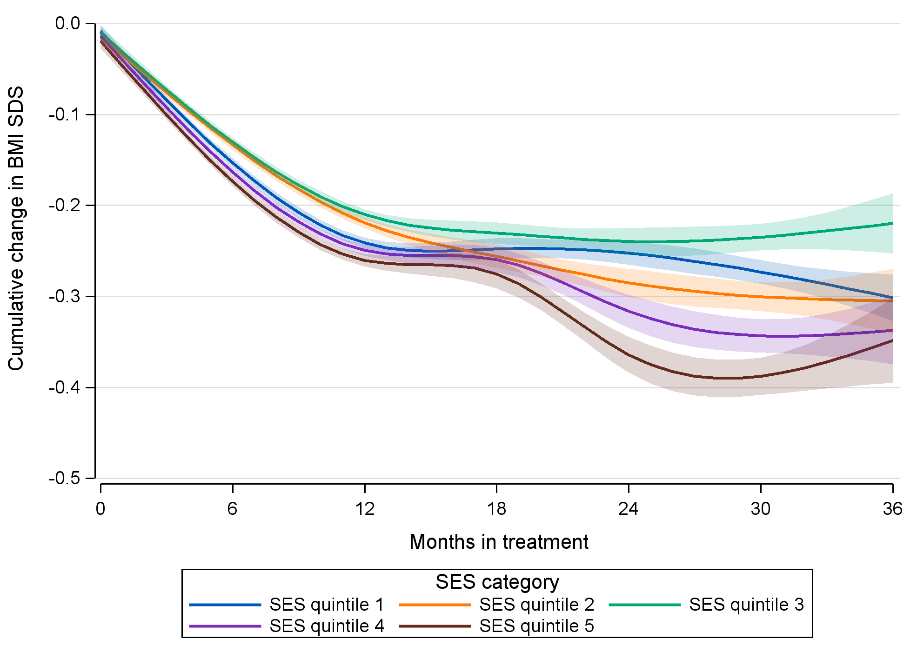


**Fig C. Sensitivity analyses, with baseline BMI SDS adjustment instead of obesity class, of change in BMI SDS from baseline over three years of treatment, shown separately for Sweden and Germany, by socioeconomic status (SES) quintile (Q1–Q5), where Q1 represents the least advantaged and Q5 the most advantaged. Estimates are derived from linear mixed-effect models with spline, including repeated measurements, and adjusted for sex, age group, BMISDS, and migration.**

Sweden Germany


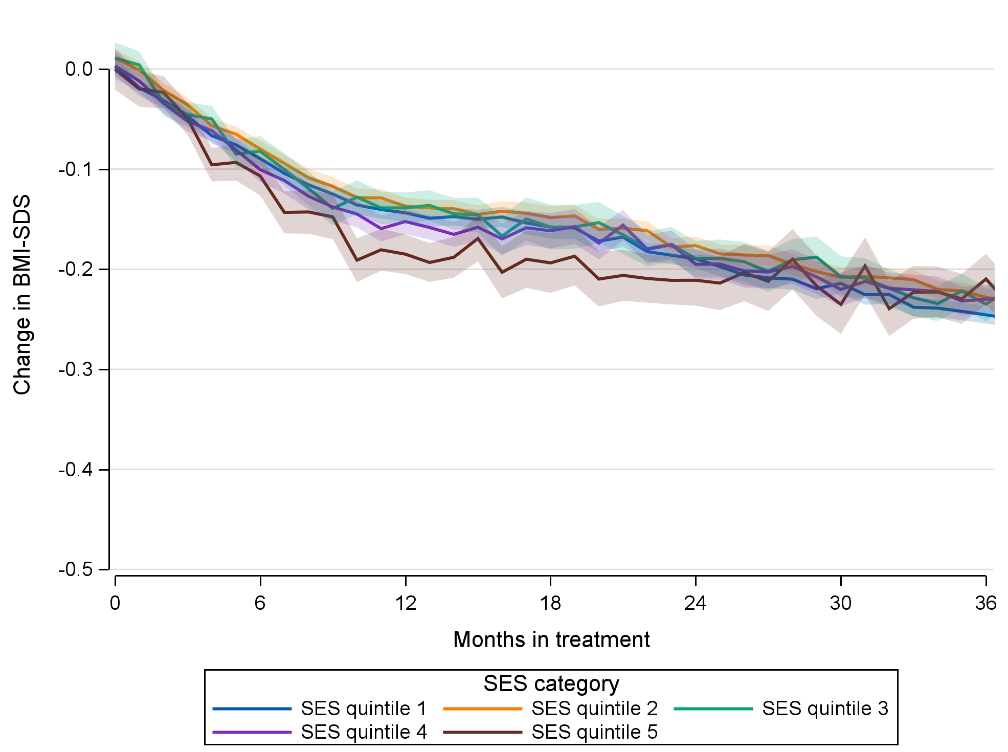

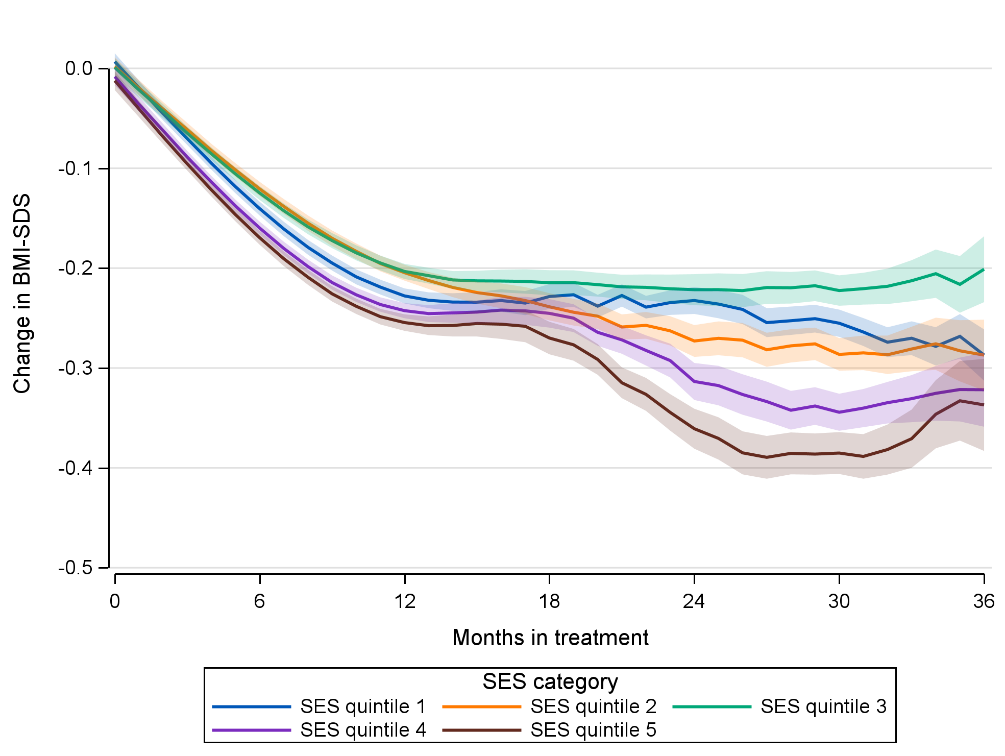


**Fig D. Sensitivity analyses, without baseline obesity class adjustment of change in BMI SDS from baseline over three years of treatment, shown separately for Sweden and Germany, by socioeconomic status (SES) quintile (Q1–Q5), where Q1 represents the least advantaged and Q5 the most advantaged. Estimates are derived from linear mixed-effect models with spline, including repeated measurements, and adjusted for sex, age group, and migration.**

Sweden Germany


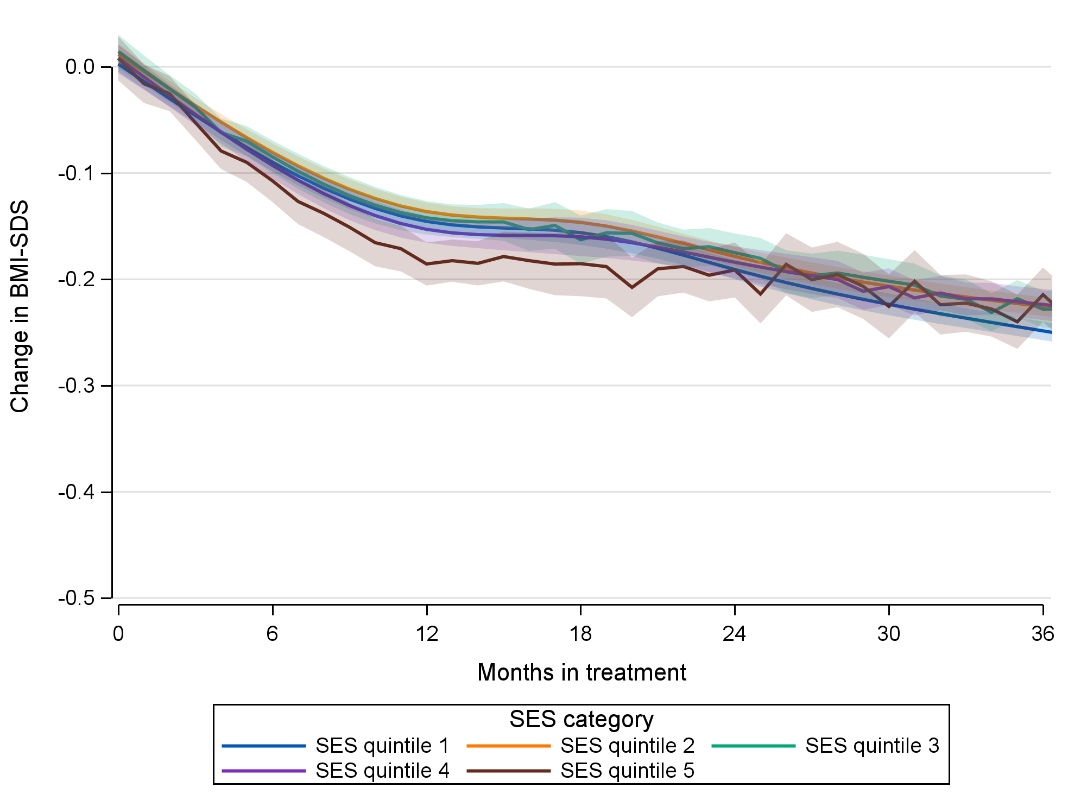

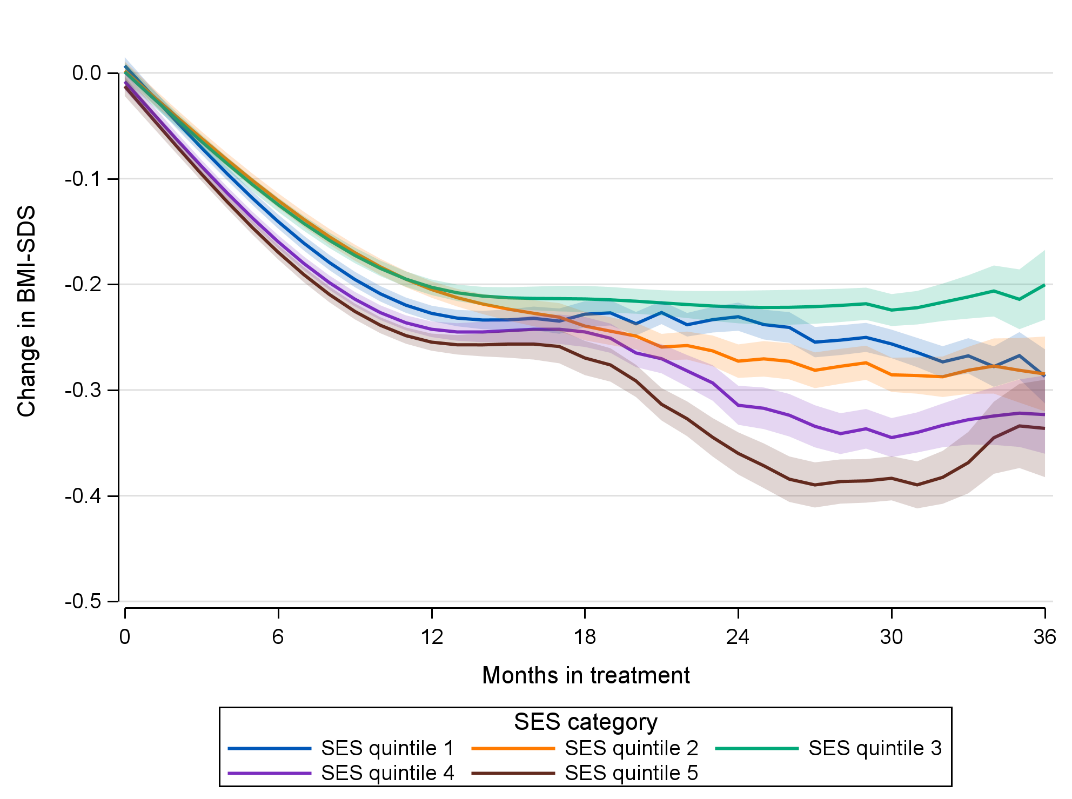


**Fig E. Probability of obesity remission over three years by SES quintiles, from Cox regression model, adjusted for sex, age group, obesity class at baseline, migration background, and country. Swe, Sweden; Ger, Germany.**
