# Supplementary material for: The association of socioeconomic status and response to pediatric health behavior and lifestyle obesity treatment in Germany and Sweden: A multiyear, two-cohort observational study
Source: PLoS Med. 2026 Jul 9;23(7):e1004909. doi: 10.1371/journal.pmed.1004909 (PMC13367900; doi:10.1371/journal.pmed.1004909)
Supplement: S1 Checklist — This checklist is licensed under a CCBY 4.0 license (https://creativecommons.org/licenses/by/4.0/deed.en). https://www.strobe-statement.org/checklists/. (PDF) [file pmed.1004909.s002.pdf]

STROBE Statement—Checklist of items that should be included in reports of ***cohort studies***

	Item No	Recommendation	Section/Paragraph
**Title and abstract**	1	(*a*) Indicate the study's design with a commonly used term in the title or the abstract	Title and Abstract
		(*b*) Provide in the abstract an informative and balanced summary of what was done and what was found	Abstract
**Introduction**			
Background/rationale	2	Explain the scientific background and rationale for the investigation being reported	Introduction
Objectives	3	State specific objectives, including any prespecified hypotheses	Last paragraph of Introduction
**Methods**			
Study design	4	Present key elements of study design early in the paper	First paragraph of the Methods
Setting	5	Describe the setting, locations, and relevant dates, including periods of recruitment, exposure, follow-up, and data collection	First and second paragraph of the Methods
Participants	6	(*a*) Give the eligibility criteria, and the sources and methods of selection of participants. Describe methods of follow-up	Second paragraph of the Methods, Figure S1, and "Data sources" section in Methods
		(*b*) For matched studies, give matching criteria and number of exposed and unexposed	N/A
Variables	7	Clearly define all outcomes, exposures, predictors, potential confounders, and effect modifiers. Give diagnostic criteria, if applicable	Section "Variables and definitions" in Methods
Data sources/ measurement	8*	For each variable of interest, give sources of data and details of methods of assessment (measurement). Describe comparability of assessment methods if there is more than one group	Section "Variables and definitions" in Methods
Bias	9	Describe any efforts to address potential sources of bias	Section "Statistical analyses" in Methods
Study size	10	Explain how the study size was arrived at	Supplementary Figure 1
Quantitative variables	11	Explain how quantitative variables were handled in the analyses. If applicable, describe which groupings were chosen and why	Section "Variables and definitions" in Methods
Statistical methods	12	(*a*) Describe all statistical methods, including those used to control for confounding	Section "Statistical analyses" in Methods
		(*b*) Describe any methods used to examine subgroups and interactions	N/A
		(*c*) Explain how missing data were addressed	Section "Statistical analyses" in Methods
		(*d*) If applicable, explain how loss to follow-up was addressed	N/A
		(*e*) Describe any sensitivity analyses	N/A

**Results**			
Participants	13*	(a) Report numbers of individuals at each stage of study—eg numbers potentially eligible, examined for eligibility, confirmed eligible, included in the study, completing follow-up, and analysed	Supplementary Figure 1
		(b) Give reasons for non-participation at each stage	Supplementary Figure 1
		(c) Consider use of a flow diagram	Supplementary Figure 1
Descriptive data	14*	(a) Give characteristics of study participants (eg demographic, clinical, social) and information on exposures and potential confounders	First paragraph of Results and Table 1
		(b) Indicate number of participants with missing data for each variable of interest	N/A Complete data
		(c) Summarise follow-up time (eg, average and total amount)	First paragraph of Results and Table 1
Outcome data	15*	Report numbers of outcome events or summary measures over time	Throughout Result section for different outcomes, Supplementary Figure 2
Main results	16	(*a*) Give unadjusted estimates and, if applicable, confounder-adjusted estimates and their precision (eg, 95% confidence interval). Make clear which confounders were adjusted for and why they were included	Figure 1, Figure S3, Table S4, Table 2
		(*b*) Report category boundaries when continuous variables were categorized	Figure 1, Figure S3, Table S4, Table 2
		(*c*) If relevant, consider translating estimates of relative risk into absolute risk for a meaningful time period	Table S4, Table 2
Other analyses	17	Report other analyses done—eg analyses of subgroups and interactions, and sensitivity analyses	Table S3, Table S5, Table S6
**Discussion**			
Key results	18	Summarise key results with reference to study objectives	First paragraph of Discussion
Limitations	19	Discuss limitations of the study, taking into account sources of potential bias or imprecision. Discuss both direction and magnitude of any potential bias	Discussion
Interpretation	20	Give a cautious overall interpretation of results considering objectives, limitations, multiplicity of analyses, results from similar studies, and other relevant evidence	Last paragraph of Discussion
Generalisability	21	Discuss the generalisability (external validity) of the study results	Second paragraph of Discussion
**Other information**			
Funding	22	Give the source of funding and the role of the funders for the present study and, if applicable, for the original study on which the present article is based	Under heading “Funding”

*Give information separately for exposed and unexposed groups.

Note: An Explanation and Elaboration article discusses each checklist item and gives methodological background and published examples of transparent reporting. The STROBE checklist is best used in conjunction with this article (freely available on the Web sites of PLoS Medicine at http://www.plosmedicine.org/, Annals of Internal Medicine at http://www.annals.org/, and Epidemiology at http://www.epidem.com/). Information on the STROBE Initiative is available at http://www.strobe-statement.org.
