# Supplementary material for: The association of socioeconomic status and response to pediatric health behavior and lifestyle obesity treatment in Germany and Sweden: A multiyear, two-cohort observational study
Source: PLoS Med. 2026 Jul 9;23(7):e1004909. doi: 10.1371/journal.pmed.1004909 (PMC13367900; doi:10.1371/journal.pmed.1004909)
Supplement: S2 File — (PDF) [file pmed.1004909.s003.pdf]

SCHEDULE 1 – PROJECT PLAN

RESEARCH PROJECT DESCRIPTION

APV – BORIS collaboration

Unravelling the Link: How Socioeconomic Status Influences Obesity Treatment Effectiveness

Background: In high-income countries, a lower socioeconomic status (SES) is often linked to higher levels of childhood adiposity.[1] Low SES seems to be associated with higher dropout rates and lower compliance to paediatric obesity treatment.[2] In one study of 172 youths, low-income was associated with less weight loss after 4 months but not after 12 months.[3] In two other studies, SES was not associated with two and three years treatment outcome, respectively.[4,5] Nevertheless, there is a remarkable lack of high-quality evidence concerning the influence of social disparities on paediatric obesity treatment efficacy.[6] One reason for this may be the intricate and multidimensional aspects of SES, coupled with the absence of a standardized definition. In research, SES is often a composite measure of education, income, and occupation.

Aim: The primary aim is to evaluate if and how SES impact paediatric obesity treatment effectiveness in two high-income European countries and if it differs over years of treatment initiation, treatment duration, by sex, age and degree of obesity. The secondary aims are to evaluate if SES affects the probability of early dropout from obesity treatment and the probability of achieving obesity remission and whether potential differences vary over years of treatment initiation, by treatment duration, sex, age and degree of obesity.

Hypothesis: We hypothesis that SES is associated with the effectiveness of obesity treatment and dropout rate from such programs. Additionally, we posit that this association is most pronounced at the onset of treatment and gradually diminishes over time. Furthermore, we propose that the impact of SES on treatment effectiveness is modified by variables such as sex, age, and the level of obesity.

Setting: Longitudinal data from the paediatric obesity treatment registers BORIS (linked with national registers) from Sweden and APV with data from Germany.

Exposure: Quintiles of SES comprising a composite measure of education, income, and occupational status. In Germany, the exposure will be based on a regional socioeconomic deprivation index at the time of treatment initiation.[7] In Sweden, SES will be individual-levelled data from national registers at the time of treatment initiation.

Primary outcome: The effectiveness of paediatric obesity treatment will be assessed based on the changes in Body Mass Index Standard Deviation Scores (BMI SDS)[8] from the initial visit up to 3 or 4 years of treatment, depending on power.

Secondary outcomes: Early dropout will be defined as treatment duration less than 6 months, and obesity remission will be defined as no longer having obesity, according to IOTF, at the last registered visit or after 3 (or 4) years of treatment, whichever comes first.

Covariates: Sex (f/m), migration background (y/n), and BMI SDS[8](continuous variable) and age at obesity treatment initiation (categorised as: 3–9, 10–13, 14–17 years), year of treatment initiation

(categorised as 2000–2010, 2011–2020), presence of comorbidity (hypertension[9], dyslipidaemia[10], prediabetes[11], elevated liver enzymes[12]).

Inclusion criteria: Patients with obesity according to IOTF[8], from 3.0 to <17.0 years of age at obesity treatment initiation are eligible for inclusion.

Exclusion criteria: Obesity associated syndromes (e.g. Prader Willi and LMBB), other genetic syndromes (e.g. Mb. Down, Turner, Fragile X, Klinefelter), insulin-dependent diabetes, treatment with systemic corticoids, Cushing's disease, hypothyroidism discovered during obesity treatment (i.e. not well controlled hypothyroidism), surgical or pharmacological obesity treatment (e.g. bariatric surgery, GLP-1 RA, orlistat and sibutramine), or missing data on exposure will be excluded.

Statistical analysis plan:

Descriptive statistics: Descriptive analyses at baseline will be performed for the whole study population as well as for socioeconomic quintiles. Demographic and clinical data will be presented as median (Q1, Q3) or mean (SD) for continuous variables and as frequencies and proportions for categorical variables. See Dummy Table 1 for example. Descriptive tables for each register may be added as supplementary information.

Primary outcome: The outcome will be analysed as a continuous variable (Δ BMI SDS) using linear mixed model approach to account for repeated measurements of BMI SDS (cumulative changes of Δ BMI SDS) over time. In the fixed-effect parts we will allow for a nonlinear effect of time using natural cubic splines with x internal knots. In addition, the fixed effect parts will also include SES, age category, sex, degree of obesity, interaction term SES*time, SES*degree of obesity, and SES*sex. The random effect parts will include time and register (BORIS or APV) as random intercepts.

If there is significant interaction term, stratified analyses will be performed. Beta coefficients representing estimated mean difference, their 95% confidence intervals, and p-values will be reported. Statistical significance will be defined as a p-value of <0.05.

Results will be illustrated graphically, e.g. ΔBMI SDS on Y-axis and months or years of follow-up on the X-axis, with the categories of SES as arms.

Secondary outcomes: Both secondary outcomes will be dichotomised, e.g. yearly drop-out or not and achieving obesity remission or not. Mixed effect logistic regression will be applied to obtain probabilities. Similar fixed and random effects as the primary outcome will be included.

Distribution of work and authorship:
Conceptualization: Emilia Hagman, Resthie Putri and Marie Auzanneau
Data curation: Emilia Hagman and Resthie Putri in Sweden, and Marie Auzanneau in Germany
Formal analysis: Germany
Methodology: Emilia Hagman, Resthie Putri and Marie Auzanneau
Project administration: Emilia Hagman
Writing – original draft: Sweden
Writing – review & editing: All contributing authors

Participating researchers from Germany:

- Marie Auzanneau, PhD, MPH
- Nicole Prinz, PhD (advisory tasks)
- Stefanie Lanzinger, PhD
- Other

Participating researchers from Sweden:

- Emilia Hagman, PhD
- Resthie Putri, MD, M Sci
- Pernilla Danielsson, PhD
- Other

In this collaboration, the first author will be Marie Auzanneau (UU) and last author will be Emilia Hagman (KI) Further, an equal number of authors per registry is aimed for.

Time plan

Task	Estimated Time or Completion date
Finalise study protocol	December 2023
Collaboration agreement signed	January 2024
Sweden preparing data for Germany (including creating a new SES variable)	January-February 2024
Sweden sending data to Germany	September 2024
Formal analyses	September - November 2024
Writing draft	November - January 2024
Final draft	January 2025
Submitting manuscript	February 2025

References

1. Vazquez CE, Cubbin C. Socioeconomic Status and Childhood Obesity: a Review of Literature from the Past Decade to Inform Intervention Research. *Curr Obes Rep.* 2020.
2. Ligthart KAM, Buitendijk L, Koes BW, et al. The association between ethnicity, socioeconomic status and compliance to pediatric weight-management interventions - A systematic review. *Obes Res Clin Pract.* 2017.
3. Davison GM, Fowler LA, Ramel M, et al. Racial and socioeconomic disparities in the efficacy of a family-based treatment programme for paediatric obesity. *Pediatr Obes.* 2021.
4. Danielsson P, Svensson V, Kowalski J, et al. Importance of age for 3-year continuous behavioral obesity treatment success and dropout rate. *Obes Facts.* 2012.
5. Braet C. Patient characteristics as predictors of weight loss after an obesity treatment for children. *Obesity (Silver Spring).* 2006.
6. Lobstein T, Neveux M, Brown T, et al. Social disparities in obesity treatment for children age 3-10 years: A systematic review. *Obesity reviews : an official journal of the International Association for the Study of Obesity.* 2021.
7. Kroll LE, Schumann M, Hoebel J, et al. Regional health differences - developing a socioeconomic deprivation index for Germany. *J Health Monit.* 2017.
8. Cole TJ, Lobstein T. Extended international (IOTF) body mass index cut-offs for thinness, overweight and obesity. *Pediatr Obes.* 2012.

9. National High Blood Pressure Education Program Working Group on High Blood Pressure in C, Adolescents. The fourth report on the diagnosis, evaluation, and treatment of high blood pressure in children and adolescents. *Pediatrics.* 2004.
10. Expert Panel on Integrated Guidelines for Cardiovascular H, Risk Reduction in C, Adolescents, et al. Expert panel on integrated guidelines for cardiovascular health and risk reduction in children and adolescents: summary report. *Pediatrics.* 2011.
11. American Diabetes A. Diagnosis and classification of diabetes mellitus. *Diabetes Care.* 2011.
12. Koutny F, Weghuber D, Bollow E, et al. Prevalence of prediabetes and type 2 diabetes in children with obesity and increased transaminases in European German-speaking countries. Analysis of the APV initiative. *Pediatr Obes.* 2020.
